# Social differentiation of the perception and human tissues donation for research purposes

**DOI:** 10.3389/fgene.2022.989252

**Published:** 2022-09-15

**Authors:** Anita Majchrowska, Michał Wiechetek, Jan Domaradzki, Jakub Pawlikowski

**Affiliations:** ^1^ Chair and Department of Humanities and Social Medicine, Medical University of Lublin, Lublin, Poland; ^2^ Institute of Psychology, The John Paul II Catholic University of Lublin, Lublin, Poland; ^3^ Department of Social Sciences and Humanities, Poznan University of Medical Sciences, Poznań, Poland

**Keywords:** human biological material, tissues donation, willingness to donate, attitudes, biomedical research

## Abstract

The willingness to donate human biological material for research purposes is shaped by socio-cultural factors; however, there is a lack of studies analysing the social perception of different human tissues, which may affect such willingness. This study aimed to distinguish different sociocultural categories of human tissues and types of potential donors based on their willingness to donate material. Quantitative research was conducted on a sample of 1,100 adult Poles representative in terms of sex, place of residence and education. According to the study, people were most willing to donate urine (73.9%), blood (69.7%), hair and tears (69.6%) and the least willing to donate post-mortem brain fragments (20%), sperm (males; 36.4%) and egg cells (females; 39.6%). A factor analysis revealed four sociocultural categories of donated tissues: irrelevant, redundant, ordinary and sensitive. Based on these sociocultural categories of tissues, four types of donors were identified: reluctant, highly cooperative, average cooperative and selectively cooperative. The willingness to donate human samples for research is shaped by the sociocultural perception of different body parts and tissues. The lower the sense of “personal relationship” with a specific type of tissue, organ or part of the body, the higher the motivation to donate such biological material for research purposes. Additionally, the willingness to donate is mostly shaped by social trust in physicians and scientists, and potential donors’ engagement in charity activities.

## Introduction

Studies on human biological material (HBM) contribute to the dynamic development of contemporary scientific research, the knowledge of genetic and environmental health determinants, the discovery of new biomarkers and drugs, and the development of personalised medicine (Hewitt, 2011; Paskal et al., 2018; Maslagova et al., 2020; Trein and Wagner, 2021). It seems that a satisfactory level of social awareness and openness to cooperation and biological material donation do not follow the dynamic development of technology and quality in HBM-based research. Although the general level of acceptance for donation of HBM for research purposes is relatively high (Goodson and Vernon, 2004; Kettis-Lindblad et al., 2006; Tupasela et al., 2011; Ahram et al., 2012; Tomlinson et al., 2015), in-depth analysis in the area of specific issues related to the donation of one’s own biological material indicates many barriers impeding the collection of a satisfactory number of samples (Gross et al., 2011; Friedman et al., 2015; Davis et al., 2019; Makhlouf et al., 2019). Therefore, the success of research on HBM depends, inter alia, on the social perception of different kinds of human tissues, and the willingness to donate HBM for research purposes. Thus, determining mental, social and cultural factors affecting individual decisions related to donation is of key significance for developing a social and individual approach in this area, and for improving the effectiveness of cooperation between researchers and donors.

While most research suggests that the public expresses predominantly positive attitudes toward the donation of HBM for research purposes, it has also identified some multifaceted barriers, including cultural beliefs about the body. There should be no surprise that cultural beliefs concerning the body and its parts shape people’s attitudes towards disease, identity, sex roles, reproduction and organ donation. While some body parts are perceived as the very essence of humanity (brain, heart, blood, eyes), others are more defined in terms of functions (hands, legs, breasts), while still others are important due to their aesthetic significance (face, skin, hands) (Ashrafian, 2007; Jacyna, 2009; Riva et al., 2011). Research also shows that one of the negative predictors for biospecimen donation may be the cultural belief in the “whole body”, which assumes that the body should remain whole at burial (Boise et al., 2017; Simon et al., 2017). Moreover, especially among the Indigenous communities, which have a strong sense of cultural connection to ancestors and traditional lands, the wholeness of the body also refers to the completeness in a social and cultural context, which means that the body should be connected to ancestral lands, and that its removal from the country is not permitted. Consequently, attempts to remove any body tissue, including DNA, for overseas analysis must undergo a special consent process (Aramoana and Koea, 2020). Nevertheless, other research suggests that cultural belief in the “sacredness of blood” may also constitute a barrier to donation (Dang et al., 2014; Kowal et al., 2015; Simon et al., 2017), especially as many people believe that blood is an important source of information about individual and one’s community.

In the review of 61 social studies, it was found that donation is a complex process that may be determined by psychosocial factors such as people’s knowledge and positive opinions of biobanks, trust toward biobanking institutions, donors’ cultural and religious beliefs, and privacy protection (Domaradzki and Pawlikowski 2019). Most studies carried out so far suggest sociodemographic determinants of the approach towards one’s own biological material. A few studies indicate that demographical variables such as sex, education level, socioeconomic status, or religion can differentiate social attitudes towards biobanking, and may influence willingness to donate. Those more favourable toward donation are middle-aged (usually 40–65 years old) persons (Prictor et al., 2020; Rahm et al., 2013) with higher education (Gaskell et al., 2013) and higher economic status, who live in urban areas and have children (Ahram et al., 2014), with a higher level of medical knowledge and earlier experiences with donations (Goodson and Vernon, 2004; Lewis et al., 2013; Pawlikowski, 2013; Ahram et al., 2014; Kauffman et al., 2016; Brall et al., 2021).

Cultural factors related to the perception of the human body and associating different parts of the human body with the essence of humanity are taken into account less often. There are suggestions that donors’ cultural and religious beliefs can shape social attitudes towards biobanking (Goodson and Vernon, 2004; Hoeyer, 2010; Lewis et al., 2013; Vaz et al., 2015; De Vries et al., 2016a; De Vries et al., 2016b; Heredia et al., 2017; Merdad et al., 2017). It is also known that being a member of ethnic minorities is associated with a lower willingness to donate (Domaradzki and Pawlikowski, 2019; Abu Farha et al., 2020).

Some studies suggest that the attitude to donation can be conditioned by the interpretation of religious dogmas determining the desired and forbidden behaviour towards one’s own body (Ahram et al., 2014; Mostafazadeh-Bora and Zarghami, 2017; Lam and McCullough, 2000; Li et al., 2019; Irving et al., 2012; Irving et al., 2014). Other cultural factors affecting the approach to the body and its parts can also play a significant role in the perception of scientific research based on the donation of an HBM’s sample and developing the willingness to donate (Dang et al., 2014; Kowal et al., 2015; Simon et al., 2017; Aramoana and Koea, 2020).

Stereotypes about one’s own body, and the organs that determine its functioning and constitute humanity, may play a significant role in the decision-making process to donate biological material. There is probably a gap in the literature regarding the connection between cultural beliefs and stereotypes about the human body, and people’s motivation to donate biological material.

This study aimed to distinguish and describe different types of potential donors based on their willingness to donate material in the Polish population. It was hypothesised that the willingness to donate is associated with differentiated social perceptions of particular types of human tissues. It was assumed that people are more open to donating tissues that are not perceived as related to human essence, personal intimacy and identity. The study is part of the project establishing the Biobanking and BioMolecular Resources Research Infrastructure in Poland (bbmri.pl) as a part of the European Research Infrastructure Consortium (BBMRI.ERIC) (Witoń et al., 2017).

## Material and methods

The research was carried out on a group of 1,100 people over 18 years of age, representing the adult population of Poland in 2019. A random-quote sampling method was used for selecting the study sample. The Polish Central Statistical office data concerning people over eighteen were used to determine the study population’s size and structure. By determining the acceptable statistical error margin of ca. 4% (for the confidence level = 0.95, distribution of samples = 0.5 and population of adult Poles amounting to 31, 512, 906 people, it was calculated that the study sample should consist of 1,100 inhabitants of Poland. The selection of respondents was calculated on the basis of them being representative of the Polish population in the following areas: sex of respondents (100% compliance with calculations based on the Local Data Bank [LDB]), age of respondents (maximum deviation of 2% from calculations based on the LDB), the number of respondents in a given voivodship calculated on the basis of the population distribution throughout the country (100% compliance with calculations based on the LDB), place of residence (maximum deviation of 1% from calculations based on the LDB), level of education (maximum deviation of 3% from calculations based on the LDB) (Table 1).

**TABLE 1:** Sample characteristics.

**Variables**	N		%
**Age (M/SD)**	(47.41/17.37)		
**Sex**	Women	575	52.3
	Men	525	47.7
**Education**	Primary or vocational	79	7.2
	Secondary	596	54.2
	High	425	38.6
**Place of living**	Village	158	14.4
	City to 50,000 residents	249	22.6
	City from 50,000 to 100,000 residents	169	15.4
	City over 100,000 residents	524	47.6
**Self-assessment of material conditions**	Very bad	21	1.9
	Bad	28	2.5
	Rather bad	183	16.6
	Rather good	602	54.7
	Good	228	20.7
	Very good	38	3.5
**Self-assessment of health conditions**	Very bad	20	1.8
	Bad	38	3.5
	Rather bad	155	14.1
	Rather good	531	48.3
	Good	283	25.7
	Very good	73	6.6

The study participants were selected with the “random route” method. Participation in the study was voluntary. Beginning at the starting point (the first house number on the selected street), the interviewer visited every third residential premise (flat/detached house) until collecting a maximum of three respondents on the given street or exhausting the pool of addresses where respondents fulfilling the study inclusion criteria could stay. The maximum number of people from one locality amounted to nine respondents in cities/towns/villages with up to 100,000 people and fifteen respondents in cities/towns with over 100,000 inhabitants. At the study recruitment stage, the response rate was 72%, which means that 28% of the invited people refused to participate in the study. Most people refused because of a lack of time, but in some cases (six people - 0.5%), the interviewees reported that the refusal was justified as “I am not interested in discussing the issue/I do not want to talk about it”.

### Data collection

The data comes from cross-sectional survey-based research carried out in 2019, using a mixed-mode survey technique comprising 96% CAPI (the default technique) and 4% PAPI. A proprietary survey questionnaire was used in the research; its correctness was verified in a pilot study.

### Measures

The willingness to donate biological material was measured with the question: How much would you be willing to donate the following samples of your biological material for scientific research purposes? The respondents were asked about the willingness to donate: urine, blood, hair, oral swabs, tears, fingernails, own tissues fragments remaining after surgery, cancer tissues fragments remaining after surgery, skin specimen, egg cell, bone marrow, semen, breastmilk, a part of brain tissue–after death. The respondents ranked their willingness to donate on a scale of 1 (definitely not) to 5 (definitely yes).

In addition to standard sociodemographic information, including age, sex, education level or place of residence, such additional factors were considered as engagement in charity activities, religiousness, financial situation, health condition, trust in other people, trust in physicians and trust in scientists.

Engagement in charity activities was measured with a single question (How often did you engage or engage in selfless, organized activities for other people (e.g. voluntary work, support groups, social campaigns, etc.) rated on a five-point scale including such answers as: never, several times in my life, from time to time, quite often, I am constantly involved.

Religiosity was measured with a single question (Please rate your religious faith) rated on a seven-point scale from 1 (I am a non-believer) to 7 (I am a deeply religious person).

Trust was measured in three aspects: trust in doctors, trust in scientists, and trust in other people. The respondents were asked to answer three questions (Please rate your trust in….) relating to a specific group on a scale from 0 (I do not trust at all) to 10 (I trust completely).

Financial situation was measured with a single question (How do you assess your own financial situation?) rated on a six-point scale from 1 (very bad) to 6 (very good). Health condition was also measured with a single question (What is your current health condition?) rated on a six-point scale from 1 (very bad) to 6 (very good).

### Statistical analysis

The results were statistically analysed using IBM SPSS 27 statistical package. The analyses were carried out in three steps. The first step was meant to check the willingness to donate specific samples. Descriptive statistics were used for this purpose (mean, standard deviation). Then, a factor analysis (Varimax) was performed, which enabled us to reduce different kinds of tissues into more general categories. The following step also involved a cluster analysis (k-means method) in differentiating the donor types for the intensity of their willingness to donate different tissue categories for research purposes. The last step of analysis included comparing the identified donor types with other variables controlled in the study. To achieve that, depending on the scale of measurement, a one-way ANOVA and Kruskal–Wallis ANOVA on ranks, including multiple comparison tests, were used. A threshold of *p* < 0.05 was assumed for statistical significance. Due to the different types of measurement scales used during the study, various types of descriptive statistics were used to present the results of specific variables. For quantitative scales, the mean and standard deviation were used, and for the rank scale, the median and quartile deviation were used. It allows to visualize the intensity of individual results and to assess their dispersion around the central point.

## Results

The results are presented in several stages. The willingness to donate specific tissues for research purposes is described at the first stage. The second one presents the result of factor analysis and tissue grouping into sizes. The third one contains descriptions of the typologies of tissue donors. Finally, the fourth part applies to the psychosocial characteristics of different types of potential donors of biological material for research purposes.

### Stage I

**FIGURE 1:**
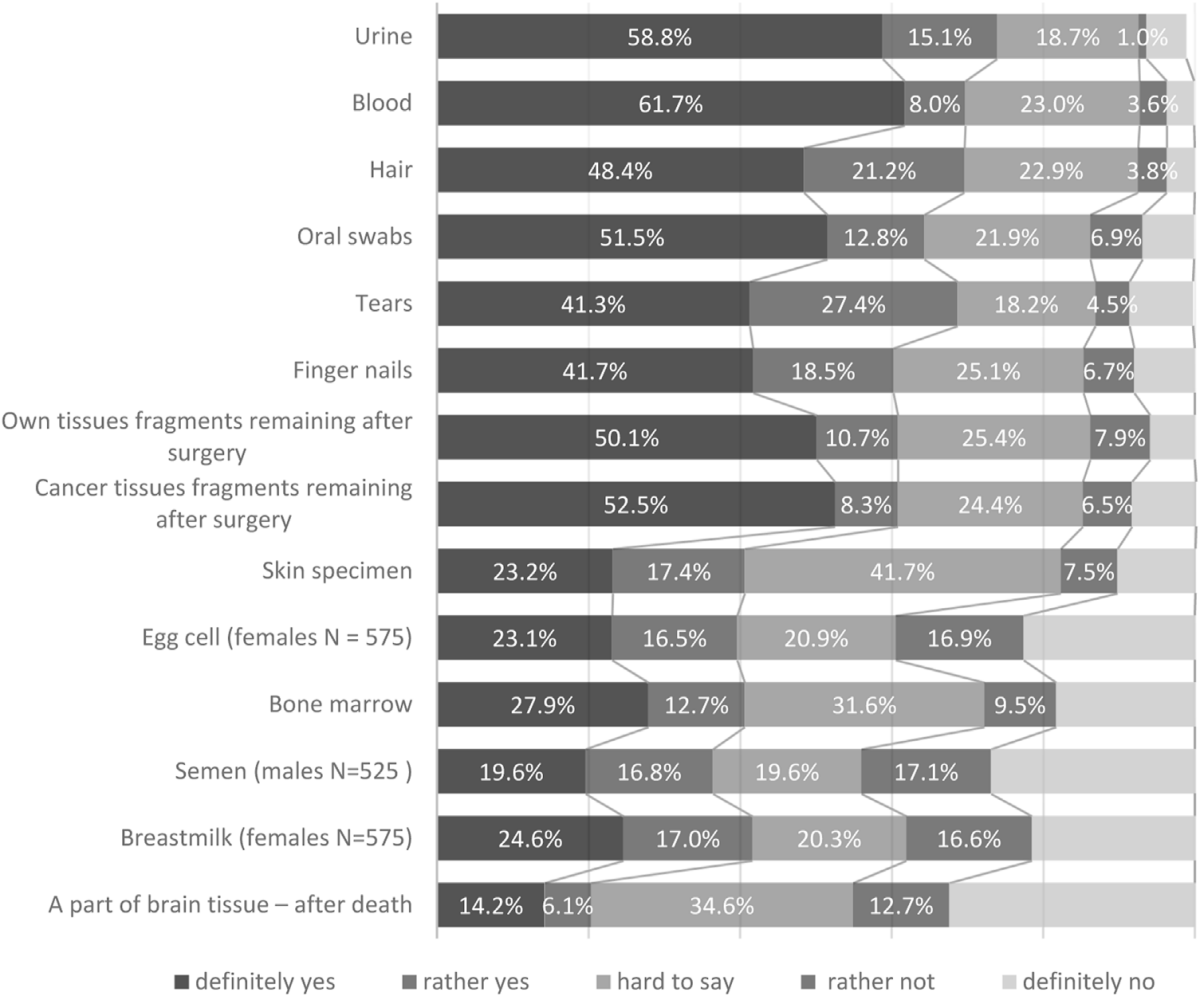
Willingness to donate human biological material.

The respondents’ willingness to donate different tissue for research purposes was identified at the first stage. The analysis reveals the respondents’ diversified attitudes depending on the tissue type. They would be most willing to donate urine (73.9%), blood (69.7%), hair (69.6%) and tears (69.0%). Respondents were the least willing to donate their biological material for research purposes in the following cases: post-mortem donation of brain fragments (20%), sperm (males; 36.4%), egg cells (females; 39.6%) and bone marrow (40.5%) (Figure 1).

Analysing the respondents’ selection median yields minor differences in ranking the preferences. The highest willingness to donate was observed for blood (4.45), urine (4.44), saliva (4.26) and nails (4.25), while the lowest one applied to the donation of parts of the brain (2.48), egg cell (females; 3.10), sperm (males; 3.10) and bone marrow (3.10).

### Stage II

The results concerning willingness to donate specific tissues (except for donating breastmilk—a category including only females) were used to carry out a factor analysis with the Varimax method (Table 2). It was meant to check if tissues could be combined into more general categories (factors/dimensions). The procedure’s outcomes have helped differentiate four factors, which explain 49.15% of the variance related to the perception of willingness to donate tissues. The factor loads in various dimensions were satisfactory, and ranged from 0.42 to 0.95. The first factor (Irrelevant) included nails and tears. The other one (Redundant) applied to brain tissue fragments (after death), own tissue fragments remaining after (past or possible) surgery, and cancer tissue fragments remaining after (past or possible) surgery. The third dimension (Ordinary) consisted of tissues most often donated for laboratory tests, including urine, blood, skin specimen, and bone marrow. The fourth dimension (Sensitive) covered sperm (males), egg cells (females) and oral swabs.

**TABLE 2:** Results of factor analysis on different types of tissues.

Type of tissue				
	**Irrelevant (factor 1)**	**Redundant (factor 2)**	**Ordinary (factor 3)**	**Sensitive (factor 4)**
Nails	0.95			
Tears	0.95			
Brain tissue fragments (after death)		0.81		
Own tissues fragments remaining after (past or possible) surgery		0.66		
*Cancer* tissues fragments remaining after (past or possible) surgery		0.45		
Urine			0.62	
Blood			0.60	
Skin specimen			0.50	
Bone marrow			0.42	
Hair^a^			-	
Semen (males)/egg cell (females)				0.67
Oral swab				0.67

a Factor loadings lower than 0.4 have been removed from the table.

### Stage III

**FIGURE 2:**
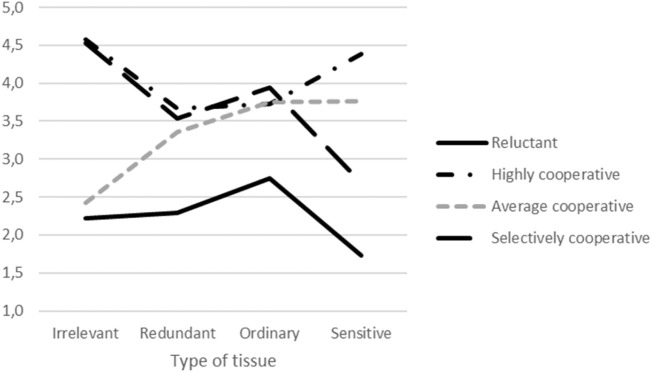
Results of the K-means cluster analysis of the results showing the willingness to donate.

Based on the factor analysis, the results for each dimension were calculated by averaging the respondents’ answers for the tissues that constituted the particular dimension. Then, the material prepared this way was used for grouping the participants using the K-means cluster analysis method. Consequently, four clusters of different potential donors types were obtained, who differed in their willingness to donate tissues for research purposes (Figure 2).

The first group (Reluctant, *n* = 65) was characterised by a low willingness to donate tissues to a biobank. Group 2 (Highly cooperative, *n* = 385) included people open to cooperation with biobanks and willing to donate all types of tissues. People demonstrating average willingness to donate their material to biobanks (Average cooperative, *n* = 294) constituted the third group. Finally, the last type of donors (Selectively cooperative, *n* = 365) were the people who declared their general willingness to donate biological material except for tissues that are culturally sensitive or might carry sensitive data (semen (males)/egg cell (females) and oral swab).

### Stage IV

The identified potential types of donors were compared for different psychosocial variables such as sex, age, education, place of residence, religiousness, financial situation, health condition, engagement in charity activities and trust in different groups (other people, scientists, physicians) (Table 3).

**TABLE 3:** Comparisons of tissue donor types for psychosocial variables.

Variable	Reluctant (1)		Highly cooperative (2)		Average cooperative (3)		Selectively cooperative (4)		F/H	p	Post hoc
	**M/Me**	**SD/Q**	**M/Me**	**SD/Q**	**M/Me**	**SD/Q**	**M/Me**	**SD/Q**			
Age	51.15	19.88	46.94	16.89	46.77	17.37	47.75	17.37	1.28	0.280	
Education	2.31	0.61	2.34	0.59	2.38	0.59	2.30	0.59	3.18	0.365	
Locality (population)	2.41	0.79	2.06	1.06	2.23	0.98	2.18	0.97	6.42	0.093	
Engagement in charity	2.11	0.72	2.30	0.75	2.48	0.78	2.45	0.85	11.79	0.008	1:4; 1:3; 2:4; 2:3
Religiousness	4.95	1.68	4.36	1.88	4.78	1.60	4.46	1.57	5.46	0.141	
Financial situation	3.80	1.14	3.92	0.86	4.05	0.90	4.09	0.84	3.77	0.010	1:3; 1:4; 2:3; 2:4; 3:2
Health condition	3.89	1.05	4.16	0.93	4.20	0.94	4.07	1.03	2.33	0.073	1:2; 1:3
Trust in other people	5.25	2.42	5.55	2.44	5.56	2.23	5.59	2.22	0.42	0.741	
Trust in physicians	5.22	2.55	6.11	2.34	5.99	2.31	6.14	2.29	3.12	0.025	1:2; 1:3; 1:4
Trust in scientists	5.62	2.65	6.41	2.33	6.63	2.21	6.70	2.26	4.62	0.003	1:2; 1:3; 1:4

Symbols: M-mean; Me–Median; SD, standard deviation; Q-quartile deviation; F- one-way (single-factor) analysis of variance; H–Kruskal–Wallis One-Way Analysis-of Variance-by-Rank.

The potential donors’ types we identified did not differ statistically for sex (χ2 = 5.05; *p* = 0.095), age, education, religiousness or trust in other people. For other controlled variables, significant statistical differences were observed. Types of potential donors differed for engagement in charity. This type of engagement occurred least often in the people least willing to donate tissues to a biobank, and most often in the respondents with average willingness to get involved in biobanks and those willing to donate their biological material to biobanks, except for sensitive tissues.

Moreover, a relationship of a similar nature was observed for the financial situation. The persons with average willingness to donate biological material and cooperate selectively reached the relatively highest scores, while the scores of the “Reluctant” group were the lowest. Statistically significant differences were also revealed for trust in physicians and scientists. The subjects in the “Highly cooperative” and “Selectively cooperative” subgroups trusted physicians the most, while those representing the Reluctant group, the least. A similar relationship was observed for trust in scientists. People who “Selectively cooperate” and those described as “Average cooperative” trust scientists the most.

## Discussion

The research has revealed that the general level of acceptance for donation of HBM for research purposes is relatively high. Similar results have been obtained in other countries (Goodson and Vernon, 2004; Kettis-Lindblad et al., 2006; Gross et al., 2011; Vaz et al., 2015; Merdad et al., 2017; Raivola et al., 2019; Mezinska et al., 2020; Brall et al., 2021). The Poles’ willingness to donate a specific type of biological material is highly compliant with the research results from other countries. This suggests that the type of biological material is among the factors determining the willingness to donate. The highest willingness was observed for the blood, urine, saliva and hair (Gross et al., 2011; Merdad et al., 2017; Khatib et al., 2021), cancer tissues, skin and kidney tissues (Vaz et al., 2015), while the lowest was observed for the eyes, brain, lungs and heart (Goodson and Vernon, 2004; Kettis-Lindblad et al., 2006; Lewis et al., 2013).

A factor analysis was performed in search of the cultural factors determining the donation preferences. It revealed the complexity of deciding to donate tissues and significant differences between the material types. The differentiated tissues were grouped into four categories. The first category included nails and tears that can be interpreted as regenerating body parts, external to the body, renewing and not carrying any essential information; hence, the feeling of personal relationship with such tissues was low (therefore they are “irrelevant” to a human person). Moreover, the donation does not entail any risk or suffering, which some studies identified as a constraint preventing material donation (Heredia et al., 2017). The second group covered tissues that can be interpreted as “unnecessary”/redundant: parts of the body donated after death (brain tissue), cancer, or own tissues remaining after surgery. The respondents do not feel attached to them; they see them as external, unsuitable or even dangerous (illness, surgery, death). The third group seems ambiguous. They are related more to the inner sphere of the organism, can carry a lot of information about us, and are important for the body’s functioning; the donation may sometimes be painful (e.g., blood and bone marrow), but it is not associated with permanent damage to the body. The last group contains samples belonging to a very personal and sensitive sphere (sexuality, oral cavity) that can be associated with sensitive data. The factor analysis indicates the respondents’ consistency in their motivation to donate tissues. A conclusion can be drawn that there is a greater or lesser willingness to donate tissues, depending on the symbolism attached to particular kinds of tissues.

Simultaneously, a higher level of social engagement and trust in physicians and scientists was identified to significantly contribute to a higher willingness to donate their biological material for scientific purposes. This may suggest that combining an altruistic approach, expressed by charity activity, with trust in medicine as the area of scientists’ and physicians’ cooperation stimulates the motivation to support science by donating one’s biological material. Moreover, it can be treated as a form of charity. Previous studies have confirmed the role of trust in physicians and medicine. They have revealed that being a patient (Johnsson et al., 2010; Gross et al., 2011) or working in the healthcare system (Gross et al., 2011) correlate with a higher willingness to donate biological material for tests. According to some research, trust is an essential determinant of the participants’ decisions to contribute to biobanks (O’Neill, 2003; Tutton et al., 2004; Hawkins and O’Doherty, 2010).

Additionally, some studies have demonstrated that a lack of familiarity with or knowledge of the donation procedure can cause anxiety and fear, and negatively influence the intention to donate. For example, some donors experience fear over the invasive nature of the sampling procedure: the puncture needle, pain during the process, sight of blood, blood flowing throughout the container (Wagner and Manolis, 2012; Lewis et al., 2013; Overby et al., 2015; Li et al., 2021). Others fear infection with HIV (Heredia et al., 2017). Consequently, such fears can negatively affect their willingness to donate (Gilchrist et al., 2019).

All in all, researchers using HBM should be aware that the human body is highly symbolic (Ashrafian, 2007; Riva et al., 2011; de Souza Dourado et al., 2018), and many of its parts are treated as precious and play important roles in various ceremonies, and for that reason must be treated and handled in a culturally appropriate way (Armoana and KoeaHoeyer, 2010;, 2020). Thus, while public attitudes toward biobanking are shaped by many sociodemographic factors that affect individuals’ willingness to donate (Kowal et al., 2015), this research suggests that cultural circumstances also influence their decisions regarding the type of tissue they are ready to donate. It also has implications for genetic research. Firstly, it shows the socio-cultural factors influencing the process of collecting various tissues from which genetic material for research purposes is obtained. Secondly, it underlines the importance of genetic and genomic information, which is socially perceived as very sensitive and personal, and may be associated with similar (or even greater) social concerns as sensitive tissues. Therefore, managing genetic and genomic information involves a lot of responsibility and ethical vigilance. Consequently, sociocultural factors should be acknowledged by individuals responsible for the organisation of research on HMB, donor recruitment, tissue collection, or information campaigns targeted at potential donors.

## Study limitations

Although this study brings new insights into the public perception of different types of human tissues and the willingness to donate HBM for research purposes, it also has some limitations. First, there was a possibility of self-selection bias in respondents who agreed to answer the survey and who were willing to provide their material and data. Thus, the willingness rates might be lower in reality than our results suggest. Second, because our research describes respondents’ opinions at a single point in time, it would be desirable to conduct a longitudinal survey study that would assess how respondents’ views develop and change over time. Third, it is possible that respondents’ attitudes and opinions differ from those who did not respond. The inability to compare the results with the results of similar studies is a serious disadvantage of this research. That is why it should be assumed that the presented analysis is explorative. Nevertheless, more in-depth research is indispensable to identify the mechanisms shaping individual decisions about own biological material donation based on cultural factors. Fourth, we did not ask respondents’ about their previous research experiences (e.g., participation in clinical trials), although such experiences might have affected their responses. Finally, as this study is based on the quantitative method only, to understand better the respondents’ motivations, opinions and willingness to donate, further in-depth studies using qualitative methods would be required.

However, despite these limitations, some advantages of this study should also be acknowledged. Most importantly, as there is a scarcity of previous work on the topic, this research fills a gap in the literature regarding the willingness to donate HBM for research purposes in Poland. Another strength of our study is the use of a national sample representing Polish residents according to the most important sociodemographic characteristics. Thus, we believe that the results reflect the attitudes and opinions of the Polish population, with a small bias. Finally, we believe that it may stimulate further research on the topic.

## Conclusion

The study results indicate a positive approach to donating one’s own biological material. However, the willingness to donate depends on the social perception of the different tissues and body parts. The lower the sense of “personal relationship” with a specific type of tissue, organ or part of the body, the higher the motivation to donate such biological material for research purposes. Additionally, the willingness to donate is mostly shaped by social trust in physicians and scientists and the potential donors’ engagement in charity activities. Therefore, information campaigns to encourage the donation of samples for research purposes should take into account sociodemographic variables, cultural beliefs about the body, and stereotypes deeply embedded in human consciousness.

## Data Availability

The raw data supporting the conclusions of this article will be made available by the authors, without undue reservation.

## Abbreviations

HBM, human biological material; LDB, Local Data Bank.
