# Supplementary material for: Social differentiation of the perception and human tissues donation for research purposes
Source: Front Genet. 2022 Sep 15;13:989252. doi: 10.3389/fgene.2022.989252 (PMC9521191; doi:10.3389/fgene.2022.989252)
Supplement: Supplementary file 1 [file DataSheet1.PDF]

1. Płeć

- ☐ Kobieta
- ☐ Mężczyzna

2. Wiek

- ☐ ………. Lat

3. Miejsce zamieszkania (województwo)

- ☐ Dolnośląskie
- ☐ Kujawsko-pomorskie
- ☐ Lubelskie
- ☐ Lubuskie
- ☐ Łódzkie
- ☐ Mazowieckie
- ☐ Mazowieckie
- ☐ Opolskie
- ☐ Podkarpackie
- ☐ Podlaskie
- ☐ Pomorskie
- ☐ Śląskie
- ☐ Świętokrzyskie
- ☐ Warmińsko-mazurskie
- ☐ Wielkopolskie
- ☐ Zachodniopomorskie

4. Wykształcenie

- ☐ Podstawowe lub zawodowe
- ☐ Średnie
- ☐ Wyższe

5. Miejsce zamieszkania (wielkość miejsca zamieszkania)

- ☐ Wieś
- ☐ Miasto do 50 tys. mieszkańców
- ☐ Miasto od 50 do 100 tys. mieszkańców

6. Jak często angażował lub angażuje się Pan/Pani w bezinteresowną zorganizowaną działalność na rzecz innych osób (np. wolontariat, grupy pomocy, grupy wsparcia, akcje społeczne, itp.)?

- ☐ Nigdy
- ☐ Kilka razy w życiu
- ☐ Od czasu do czasu
- ☐ Dość często
- ☐ Jestem stale zaangażowany

7. **Jak ocenia Pan/Pani własną sytuację materialną?**

- Bardzo zła
- Zła
- Raczej zła
- Raczej dobra
- Dobra
- Bardzo dobra

8. **Jaki ocenia Pan/Pani swój aktualny stan zdrowia?**

- Bardzo zły
- Zły
- Raczej zły
- Raczej dobry
- Dobry
- Bardzo dobry

9. **W jakim stopniu byłby Pan/Pani skłonny oddać do badań naukowych następujące próbki własnego materiału biologicznego?**

	**Zdecydowanie nie**	**Raczej nie**	**Trudno powiedzieć**	**Raczej tak**	**Zdecydowanie tak**
Krew					
Mocz					
Szpik kostny					
Włosy					
Wycinek skóry					
Łzy					
Paznokcie					
Wymaz z jamy ustnej					
Fragmenty nowotworu pozostałe po operacji (przebytej lub ewentualnej)					
Fragmenty własnych tkanek pozostałe po operacji (przebytej lub ewentualnej)					
Nasienie (mężczyźni) / komórka jajowa (kobiety)					
Mleko (kobiety)					
Fragmenty tkanki mózgowej po śmierci					

10. **Proszę ocenić Pana/Pani zaufanie do innych ludzi**

**Zupełnie nie ufam**	**0**	**1**	**2**	**3**	**4**	**5**	**6**	**7**	**8**	**9**	**10**	**W pełni ufam**

11. Proszę ocenić Pana/Pani zaufanie do lekarzy

Zupełnie nie ufam	0	1	2	3	4	5	6	7	8	9	10	W pełni ufam

12. Proszę ocenić Pana/Pani zaufanie do naukowców

Zupełnie nie ufam	0	1	2	3	4	5	6	7	8	9	10	W pełni ufam

13. Proszę ocenić Pana/Pani wiarę religijną

Jestem osobą niewierzącą	1	2	3	4	5	6	7	Jestem osobą głęboko wierzącą
